# Interdependence of Sectors of Economic Activities for World Countries from the Reduced Google Matrix Analysis of WTO Data

**DOI:** 10.3390/e22121407

**Published:** 2020-12-13

**Authors:** Célestin Coquidé, José Lages, Dima L. Shepelyansky

**Affiliations:** 1 Institut UTINAM, OSU THETA, Université Bourgogne Franche-Comté, CNRS, 25000 Besançon, France; celestin.coquide@utinam.cnrs.fr; 2 Laboratoire de Physique Théorique, IRSAMC, Université de Toulouse, CNRS, UPS, 31062 Toulouse, France; dima@irsamc.ups-tlse.fr

**Keywords:** World Trade Organization, networks, Google matrix, Markovian process, PageRank

## Abstract

We apply the recently developed reduced Google matrix algorithm for the analysis of the OECD-WTO World Network of Economic Activities. This approach allows to determine interdependencies and interactions of economy sectors of several countries, including China, Russia and the USA, properly taking into account the influence of all the other world countries and their economic activities. Within this analysis, we also obtain the sensitivity of EU countries’ economies to the petroleum activity sector. We show that this approach takes into account the multiplicity of economical interactions between countries and activity sectors, thus providing a richer analysis compared to the usual export-import analysis.

## 1. Introduction

The statistical data of UN COMTRADE [1] and the World Trade Organization (WTO) Statistical Review 2018 [2] demonstrate all the complexity of the international trade and the economic relations between world countries. The world economy and the international trade are mutually interacting which makes the analysis of their development very important but also complicated [3]. Thus, developed advanced mathematical tools are required for the scientific analysis of such complex systems. Usually, their analysis uses the matrix tools of input-output transactions broadly applied in economy and initiated by the fundamental works of Leontief [4,5]. More recent developments are described in [6].

The complex networks constitute a domain of research which emerged in the last two decades alongside with the development of the modern society generating enormous amount of communication including the World Wide Web (WWW), Wikipedia, Facebook, Twitter (see e.g., [7]). The PageRank algorithm, developed by Brin and Page in 1998 [8] to retrieve information from the WWW, was at the mathematical foundation of the Google search engine (see e.g., [9]). This algorithm constructs the Google matrix *G* describing Markov chain transitions between the nodes of the WWW network and allows it to rank billions of web pages of the WWW. The efficient applications of the Google matrix analysis to various directed networks have been demonstrated in [10].

The application of the Google matrix approach to the World Trade Network (WTN) was pushed forward in [11,12] using the UN COMTRADE database [1] which contain information for almost 50 years of world trade. In addition to the PageRank algorithm, it was shown that the analysis of the WTN with the CheiRank algorithm [13,14], assuming inverted links, plays also an important role for the study of the world trade. Indeed, the PageRank probabilities of nodes are on average proportional to the number of ingoing links characterizing the import capabilities of the economic actors while the CheiRank probabilities are on average proportional to the number of outgoing links, thus, characterizing export capabilities [11,12,15]. Since both export and import have to be taken into account to correctly describe the world trade, this clearly shows the importance of the combined PageRank-CheiRank analysis. A peculiar feature of the Google matrix approach is the democratic treatment of world countries. They are treated independently of their richness which is different from the usual import and export ranking. The contributions of the various exchanges of products are taken to be proportional to their trade volume.

While the UN COMTRADE database contains an enormous amount of information for all the UN countries with thousands of exchanged products, it records gross flows of commodities and services and consequently counts the value of a product several times as it crosses borders. In the present study, we use the OECD-WTO Trade in Value Added (TiVA) database, which is an Input-Output database, containing net flows of products excluding the contributions of intermediate inputs from upstream industries. The Google matrix analysis of the World Network of Economic Activities (WNEA) constructed from the OECD-WTO TiVA has been reported in [16]. The approach developed in the OECD-WTO WNEA (2013) incorporates naturally the economic flows between activity sectors [16] which by construction were absent from the UN COMTRADE based WTN [11,12].

Hence, the new important element of the WNEA is the presence of direct interactions between the economical sectors. It is interesting to know what are the differences and the similarities of the economic transfers between the sectors of a specific country taking into account their exchanges with the sectors of the other world countries. To obtain the interactions between sectors of a given country, one should take into account their direct interactions (direct links) but also all the indirect pathways of product transfers throughout the multiplicity of exchanges with the rest of the world. The most appropriate mathematical tool for the extraction of such direct and indirect interactions is the reduced Google matrix algorithm (REGOMAX) [17]. The efficiency of this approach has been demonstrated in various field such as Wikipedia networks (e.g., interactions between politicians [18], ranking of and interactions between world universities [19], interactions between the largest world banks [20]) and biological networks encoding protein-protein interactions [21]. Recently, the REGOMAX analysis of the UN COMTRADE database allowed to obtain the influence of the petroleum and the gas trades on the economy of the EU countries [22]. Here, we use the REGOMAX approach to obtain the interdependence of economic sectors for world countries from the OECD-WTO TiVA database (WTO data) already studied in [16]. We note that other similar input-output databases exist such as the World Input-Output Database (WIOD) [23]. In principle, the reduced Google matrix analysis can be also applied to these databases and it would be interesting to probe the similarity of the main conclusions of the present study with the ones we could obtained with, e.g., WIOD.

Previous investigations of the World Trade Network data sets have been realized (see e.g., [24,25,26,27,28,29]), however the main different feature with our approach is the use of the Google matrix methods which characterize both import and export flows taking into account the whole transfer chain between the nodes of the global network. The analysis of both the import and the export directions is rather rare, see e.g., [30] where the hubs and authorities of the WTN have been studied, but we think that the Google matrix analysis using the PageRank, the CheiRank and the REGOMAX tools characterizes the economical activities in at a deeper and a more detailed level. The matrix analysis of the financial risks already demonstrated its efficiency for undirected flows [31,32,33]. However, the financial and trade flows are directional, and thus, we hope that the Google matrix tools used here will find further useful applications for the study of financial flows and the understanding of economy complexity. The recent studies of directed interbank interactions [34,35] indicate possible interesting applications of the Google matrix analysis to the study of financial flows between banks. Recent studies [36,37,38] also measure the PageRank centrality of production networks and Global Value Chains. Here, we go well beyond this sole measure: (1) we take into account the full Google matrix description of the problem as we build two Google matrices. A first one associated to the economical network with the links giving the direction of the flow of goods, and the second one with inverted links. This description allows us to determine a PageRank-CheiRank balance for economical sectors and/or for countries which carries more information than the usual accounting import-export balance [22] as it takes into account the complexity of the entanglement of countries and economical sectors. (2) Moreover, we apply the REGOMAX algorithm allowing to, for example, extract the direct and the effective indirect links of a given country economical sectors taken into account the complete information embedded in the global network describing the complex exchanges between all the sectorial activities of all the countries.

We suppose that the REGOMAX algorithm, developed from the physical problems of quantum scattering [17], can become a useful tool for research in the field of econophysics [39]. We note that the concept of entropy characterizes a possible information amount stored in a system [40]. In a steady-state (like in a thermal equilibrium), the system is characterized by a certain thermal like distribution when all the information flows inside the system are equilibrated. The Google matrix elements describe the transition probabilities between system’s sites and the information flows between them. The stationary probabilities over the sites are given by the PageRank and the CheiRank vectors. Thus, the Google matrix analysis provides an extension of entropy-type description to network systems.

## 2. Methods and Data Description

### 2.1. WNEA Data Sets

As in [16], we use the data available from the OECD-WTO TiVA database released in May 2013 which covers the years 1995, 2000, 2005, 2008, 2009. The network contains $N_{c}=58$ world countries (57 plus 1 for the Rest Of the World ROW) given in Table 1 in [16]. It contains the main world countries. We do not reproduce this list here since we concentrate our analysis only on several leading countries with the main emphasis on USA, Russia and China. We use for countries ISO-3166-1 alpha-3 code available at Wikipedia. There are also $N_{s}=37$ sectors of economic activities given in Table 1. The sectors are classified according to the International Standard Industrial Classification of All Economic Activities (ISIC) Rev.3 described in [1] and in Wikipedia. We take into account all the 37 sectors, noting that the sectors s=1,⋯,21 represent production activities while s=22,⋯,37 represent service activities. We concentrate our analysis on sectors s=1,⋯,21. The total size of the Google matrix is $N=N_{c}N_{s}=58\times 37=2146$. The main analysis is presented for year 2008. Additional data for other years are available upon request. In addition, all the OECD-WTO TiVA network data are available upon request [41].

### 2.2. Google Matrix Construction for WNEA

In the following, we use the approach developed in [12,16] to construct the Google matrix of the economical transfers between the activity sectors of the different countries. We keep the notations used in [16].

From the WTO data, we construct the matrix $M_{cc^{\prime },ss^{\prime }}$ of money transfer between nodes expressed in USD of the current year

(1) $$M_{cc^{\prime },ss^{\prime }}=\mathrm{transfer}\;\mathrm{from}\;\mathrm{country}\;c^{\prime },\mathrm{sector}\;s^{\prime }\mathrm{to}\;\mathrm{country}\;c,\;\mathrm{sector}\;s.$$

Here the country indexes are $c,c^{\prime }\in \left\{1,\ldots ,N_{c}\right\}$ and the activity sector indexes are $s,s^{\prime }\in \left\{1,\ldots ,N_{s}\right\}$ with $N_{c}=58$ and $N_{s}=37$. Here, each node represents a pair of country and activity sector. A link gives the transfer a sector of one country to another sector of another country. We construct the matrix $M_{cc^{\prime },ss^{\prime }}$ from the TiVA Input/Output tables using the transposed representation so that the volume of the products or the sectors flows in a column from line to line; for a given country *c* we exclude possible exchanges (c,s)→(c,s) from a sector *s* to itself. The matrix construction of $M_{cc^{\prime },ss^{\prime }}$ highlights the trade exchange flows intra- and inter-countries.

The Google matrices *G* and $G^{*}$ are N×N matrices with real non-negative elements defined as

(2) $$G_{ij}=\alpha S_{ij}+\left(1-\alpha \right)v_{i},\;\mathrm{and},\;{G^{*}}_{ij}=\alpha {S^{*}}_{ij}+\left(1-\alpha \right)v_{i}^{*},$$

where $N=N_{c}\times N_{s}$, α is the damping factor (0<α<1), and v is a positive column vector called personalization vector with the normalization $\sum_{i}v_{i}=1$ [9,12]. We note that the usual Google matrix corresponds to a personalization vector with $v_{i}=1/N$. Here as in [11,12], we fix α=0.5 noting that a variation of α in a range 0.5–0.9 does not significantly affect the probability distributions of the PageRank and the CheiRank vectors [9,10,11]. The personalization vector is taken from the vector representing the exchange weight of each sector as it is described in [16] (for the multiproduct WTN the same choice of this vector is described in [12,22]). As in [12,16], we call this approach the Google Personalized Vector Method (GPVM).

The matrices *S* and $S^{*}$ are built from money matrices $M_{cc^{\prime },ss^{\prime }}$ as

(3) $$\begin{array}{ccc} S_{i,i^{\prime }} & = & \left\{\begin{array}{cc} M_{cc^{\prime },ss^{\prime }}/V_{c^{\prime }s^{\prime }}^{*} & \mathrm{if}\;V_{c^{\prime }s^{\prime }}^{*}\neq 0\\ 1/N & \mathrm{if}\;V_{c^{\prime }s^{\prime }}^{*}=0 \end{array}\right.\\ S_{i,i^{\prime }}^{*} & = & \left\{\begin{array}{cc} M_{c^{\prime }c,s^{\prime }s}/V_{c^{\prime }s^{\prime }} & \mathrm{if}\;V_{c^{\prime }s^{\prime }}\neq 0\\ 1/N & \mathrm{if}\;V_{c^{\prime }s^{\prime }}=0 \end{array}\right. \end{array}$$

where $i^{\left(\prime \right)}=s^{\left(\prime \right)}+\left(c^{\left(\prime \right)}-1\right)N_{s}\in \left\{1,\cdots ,N\right\}$. We have also defined $V_{cs}=\sum_{c^{\prime }s^{\prime }}M_{cc^{\prime },ss^{\prime }}$ and $V_{cs}^{*}=\sum_{c^{\prime }s^{\prime }}M_{c^{\prime }c,s^{\prime }s}$ which are the total volume of import and export for the sector *s* of country *c*. The sum of the elements of each column of *S* and $S^{*}$ is normalized to unity and *G*, $G^{*}$, *S*, and $S^{*}$ belong to the class of Google matrices. The import properties are characterized by *S* and *G*, and export properties by $S^{*}$ and $G^{*}$. Let us note that the starred matrices, $G^{*}$ and $S^{*}$, are built in the same manner as the other matrices, *G* and *S*, but from the network for which all the directions of the links have been inverted. Consequently, the starred matrices, $G^{*}$ and $S^{*}$ are build from the transpose of the money matrix *M* (1).

The PageRank and CheiRank vectors are right eigenvectors of the matrices *G* and $G^{*}$ with the eigenvalue λ=1. Their components are positive nonzero real numbers and their sum is normalized to unity. The components give the probabilities to find a random seller (surfer) on a given node after a long walk over the network. The PageRank index *K* and the CheiRank index $K^{*}$ are defined by the components of the PageRank vector *P* and the CheiRank vector $P^{*}$ sorted by descending order, P(K)≥P(K+1) and $P^{*}\left(K^{*}\right)\geq P^{*}\left(K^{*}+1\right)$ with $K,K^{*}=1,\cdots ,N$. Since we have countries *c* and economic sectors *s*, it is convenient to use two indexes probabilities $P_{cs}$ and $P_{cs}^{*}$ with 1≤c≤58 and 1≤s≤37. The sum over all the sectors gives the probabilities $P_{c}$ and $P_{c}^{*}$ for each country *c*.

### 2.3. Reduced Google Matrix for WNEA

The REGOMAX algorithm, proposed in [17], is described in detail in [18]. Here we give the main elements of this method keeping the notations of [18,22].

The reduced Google matrix $G_{R}$ is constructed for a selected subset of $N_{r}$ nodes. The construction is based on concepts of scattering theory used in different fields including mesoscopic physics, nuclear physics, and quantum chaos. It captures, in a matrix of size $N_{r}\times N_{r}$, the full contribution of direct and indirect pathways existing in the global network of *N* nodes between the $N_{r}$ selected nodes of interest. The PageRank probabilities of the $N_{r}$ nodes are the same as for the global network with *N* nodes up to a global constant factor taking into account that the sum of PageRank probabilities over $N_{r}$ nodes is unity. The (i,j)-element of $G_{R}$ can be interpreted as the probability for a random seller (surfer) starting at node *j* to arrive in node *i* using direct and indirect interactions. Indirect interactions refer to pathways composed in part with nodes different from the $N_{r}$ ones of interest. The computation steps of $G_{R}$ offer a decomposition of $G_{R}$ into matrices that clearly distinguish direct from indirect interactions: $G_{R}=G_{\mathrm{rr}}+G_{\mathrm{pr}}+G_{\mathrm{qr}}$ [18]. Here, the $G_{\mathrm{rr}}$ matrix is generated by the direct links between selected $N_{r}$ nodes in the global *G* matrix with *N* nodes. The $G_{\mathrm{pr}}$ matrix is usually rather close to a matrix for which each column reproduces the PageRank vector $P_{r}$ associated to the $N_{r}$ nodes of interest. Due to that, $G_{\mathrm{pr}}$ does not bring much information about direct and indirect links between selected nodes. The interesting role is played by $G_{\mathrm{qr}}$. It takes into account all the indirect links between the $N_{r}$ selected nodes appearing due to the myriads of pathways passing via the rest of the $N-N_{r}≃N$ nodes of the global network (see [17,18]). The matrix $G_{\mathrm{qr}}=G_{\mathrm{qrd}}+G_{\mathrm{qrnd}}$ has a diagonal part ($G_{\mathrm{qrd}}$) and a non-diagonal part ($G_{\mathrm{qrnd}}$). The $G_{\mathrm{qrnd}}$ matrix describes indirect interactions between different nodes. The explicit formulas of the mathematical and numerical computation methods of all three matrix components of $G_{R}$ are given in [17,18,22].

### 2.4. Sensitivity of the Economy Balance

As in [22], within the REGOMAX approach, we determine the whole economy balance of a given country with PageRank and CheiRank probabilities as $B_{c}=\left(P_{c}^{*}-P_{c}\right)/\left(P_{c}^{*}+P_{c}\right)$. The sensitivity of the country *c* economy balance $B_{c}$ to the price of, e.g., the sector *s* of petroleum can be obtained: (1) by changing the corresponding money volume flow related to this sector multiplying it by (1+δ), (2) by recomputing all the rank probabilities, and then 3- by computing the derivative $D\left(s\to c\right)=dB_{c}/d\delta$. This approach was explained and used in [12,22].

We can also use the same procedure to determine, for a given country, the sensitivity of its economy sector balance $B_{cs}=\left(P_{cs}^{*}-P_{cs}\right)/\left(P_{cs}^{*}+P_{cs}\right)$ to the price variation of the petroleum sector *s*. Then the sensitivity of a sector $s^{\prime }$ of a given country *c* to another sector *s* is defined as $D\left(s\to cs^{\prime }\right)=dB_{cs^{\prime }}/d\delta$.

The usual export-import description of economic activities (and also trade exchange) between countries takes into account only direct seller-buyer interactions. In contrast, the Google matrix approach takes into account all the chains of transactions between countries which in a steady-state limit are characterized by the PageRank and CheiRank vectors. It has been already shown that compared to the simple import-export description the Google matrix analysis gives a complementary more detailed and deep description of interactions between countries and sectors of economic activity [12,16,22]. This provides an additional relevant characterization of these interactions for policy makers allowing a better understanding of non obvious indirect economic ties between countries. As an example, our approach can be used by policy makers as a tool to contain economical crisis contagion [15].

## 3. Results

Hereafter, the economical sectors are designated by the short names given in the second column of the Table 1. For the sake of clarity, these sector short names are printed in boldface.

### 3.1. Interdependence of the USA Economy Sectors

**Figure 1:**
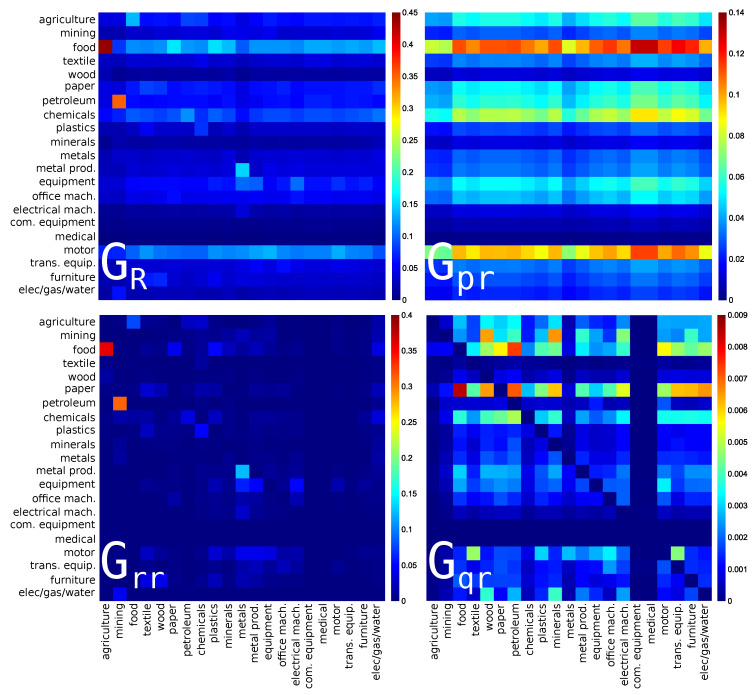
Density plot of reduced Google matrix for import or PageRank direction: $G_{R}$ (**top left**), $G_{\mathrm{pr}}$ (**top right**), $G_{\mathrm{rr}}$ (**bottom left**) and $G_{\mathrm{qr}}$ without diagonal elements (**bottom right**). The matrices are computed for a set of reduced nodes composed of $N_{r}=21$ sectors (s=1,⋯,21) of USA for the year 2008. The corresponding matrix weights are: $W_{\mathrm{pr}}=0.813817$, $W_{\mathrm{rr}}=0.155258$, $W_{\mathrm{qr}}=0.030925$ and $W_{\mathrm{qrnd}}=0.027383$. For each panel, each cell corresponds to a given value of the Google matrix component ($G_{R}$, $G_{\mathrm{pr}}$, $G_{\mathrm{rr}}$, or $G_{\mathrm{qr}}$), the colorbar gives the correspondence between matrix elements values and colors (from blue for 0 to red for the maximum). This value characterizes the intensity of the interaction between two economical sectors. The direction of the interaction is from bottom to left.

**Figure 2:**
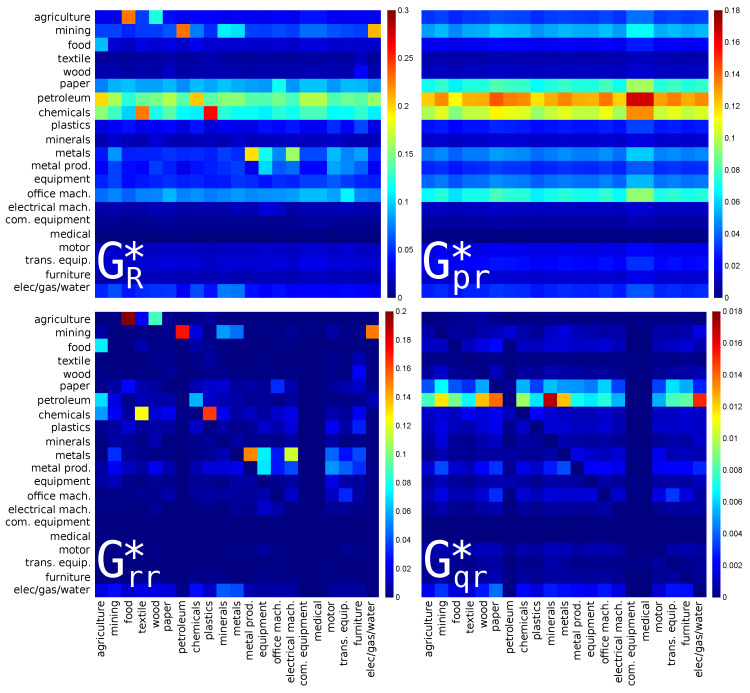
Density plot of reduced Google matrix for export or CheiRank direction: $G_{R}^{*}$ (**top left**), $G_{\mathrm{pr}}^{*}$ (**top right**), $G_{\mathrm{rr}}^{*}$ (**bottom left**) and $G_{\mathrm{qr}}^{*}$ without diagonal elements (**bottom right**). The matrices are computed for a set of reduced nodes composed of $N_{r}=21$ sectors (s=1,⋯,21) of the USA for the year 2008. The corresponding matrix weights are: $W_{\mathrm{pr}}^{*}=0.78968$, $W_{\mathrm{rr}}^{*}=0.18289$, $W_{\mathrm{qr}}^{*}=0.02743$ and $W_{\mathrm{qrnd}}^{*}=0.02554$. For each panel, each cell corresponds to a given value of the Google matrix component ($G_{R}^{*}$, $G_{\mathrm{pr}}^{*}$, $G_{\mathrm{rr}}^{*}$, or $G_{\mathrm{qr}}^{*}$), the colorbar gives the correspondence between matrix elements values and colors (from blue for 0 to red for the maximum). This value characterizes the intensity of the interaction between two economical sectors. The direction of the interaction is from bottom to left.

The reduced Google matrices $G_{R}$ and $G_{R}^{*}$ and their three matrix components for PageRank and CheiRank algorithms with $N_{r}=21$ sectors of USA economy activity (s=1,...,21 in Table 1) are shown in Figure 1 and Figure 2 respectively. As shown in [18,22], it is useful to characterize each matrix component by their weights $W_{\mathrm{pr}}$, $W_{\mathrm{rr}}$, $W_{\mathrm{qr}}$ (and $W_{\mathrm{qrnd}}$) corresponding to $G_{\mathrm{pr}}$, $G_{\mathrm{rr}}$, $G_{\mathrm{qr}}$ (and $G_{\mathrm{qrnd}}$). For each component, the weight is defined as the sum of all the matrix elements divided by the matrix size, i.e., $N_{r}$. By definition $W_{\mathrm{pr}}+W_{\mathrm{rr}}+W_{\mathrm{qr}}=1$. For the USA, the matrix weights are given in the captions of Figure 1 and Figure 2. For Wikipedia networks (see, e.g., [18,19,20]), one usually has the weights $W_{\mathrm{pr}}\approx 0.95$ and $W_{\mathrm{rr}}\approx W_{\mathrm{qr}}\approx 0.025$ [18]. Here the situation is more similar to the WTN case [22] where the weight of $W_{\mathrm{qr}}$ remains rather small while $W_{\mathrm{rr}}$ is by a factor of 3–10 larger than in Wikipedia. As for the WTN [22], we attribute this to a significantly larger number of links per node in the WTN and WNEA global networks in comparison with Wikipedia networks.

The strongest matrix elements in $G_{R}$ show the interdependence of sectors of USA for import or PageRank direction (see Figure 1). Here, we see the dominance of the interaction from the **agriculture** sector (s=1) to the **food** sector (s=3). Indeed, the agricultural activity produces food used by all the people that makes this link so strong. Another strong link is from **mining** (s=2) to **petroleum** (s=7). Indeed, coke and petroleum are produced by mining and they play an important role in the USA economy. The third link by strength is from **metals** (s=11, manufacture of basic metals) to **metal prod.** (s=12, manufacture of fabricated metal products) that is also very natural. These three links are also well present among direct links in $G_{\mathrm{rr}}$ that corresponds to the importance of direct links in the WNEA discussed above. Interestingly, other links, from **paper** (paper and paper product) and from **plastics** (rubber and plastics products) to **food**, which are not so strong in the direct matrix component $G_{\mathrm{rr}}$ are enhanced in $G_{R}$, illustrating the role of indirect interactions. Indeed, the food products industry uses products of paper and plastic industries for packaging.

Among the $G_{\mathrm{pr}}$ matrix components, there are three dominant horizontal lines for **food**, **motor** (motor vehicles), and **chemicals**, which are pointed by the majority of the sectors. These three sectors, which are major sector activities using products of many others, are at the top 3 PageRank positions of USA sectors.

The $G_{\mathrm{qr}}$ matrix components highlight hidden links between USA economic sectors. Among the most pronounced hidden interactions in $G_{\mathrm{qr}}$ we note that: **food** is pointing to **paper**, **petroleum** is pointing to **food** and **paper**. It is clearly understandable that food and paper industries indirectly use petroleum products. Concerning the **food** to **paper** link, according to $G_{\mathrm{rr}}$, the food industry directly points to agriculture, and of course the paper industry uses silviculture. This is one of the many possible indirect paths linking **food** to **paper**.

For the export or CheiRank direction, the results are shown in Figure 2. Here, the strongest links are from **plastics** (s=9, manufacture of rubber and plastics products) to **chemicals** (s=8, manufacture of chemicals), from **textile** (s=4, manufacture of textiles) to **chemicals** (s=8), and from **petroleum** to **mining**. Here again, the dominant contribution is given by $G_{\mathrm{rr}}^{*}$ but the strength of the final amplitudes is slightly corrected by $G_{\mathrm{pr}}^{*}$ and $G_{\mathrm{qr}}^{*}$ contributions which mainly highlight the fact that the petroleum and chemistry industries are the main suppliers of the other economic activity sectors.

The amplitudes of all matrix elements of $G_{R}$ for the USA, Russia (RUS), and China (CHN) and for the different years are available upon request.

### 3.2. Interdependence of the Russian Economy Sectors

**Figure 3:**
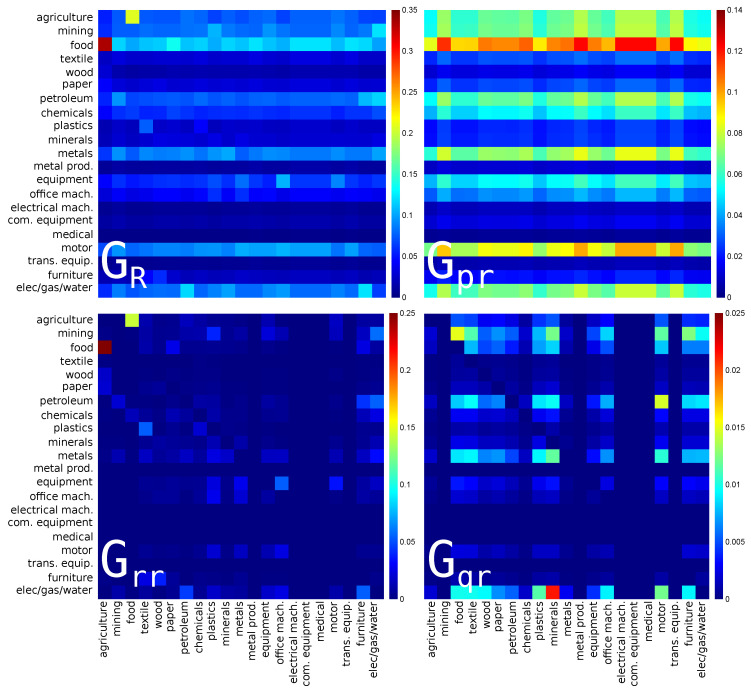
The same as in Figure 1 for Russia (RUS) in 2008. The corresponding matrix weights are: $W_{\mathrm{pr}}=0.851677$, $W_{\mathrm{rr}}=0.112809$, $W_{\mathrm{qr}}=0.035514$ and $W_{\mathrm{qrnd}}=0.033682$. For each panel, each cell corresponds to a given value of the Google matrix component ($G_{R}$, $G_{\mathrm{pr}}$, $G_{\mathrm{rr}}$, or $G_{\mathrm{qr}}$), the colorbar gives the correspondence between matrix elements values and colors (from blue for 0 to red for the maximum). This value characterizes the intensity of the interaction between two economical sectors. The direction of the interaction is from bottom to left.

**Figure 4:**
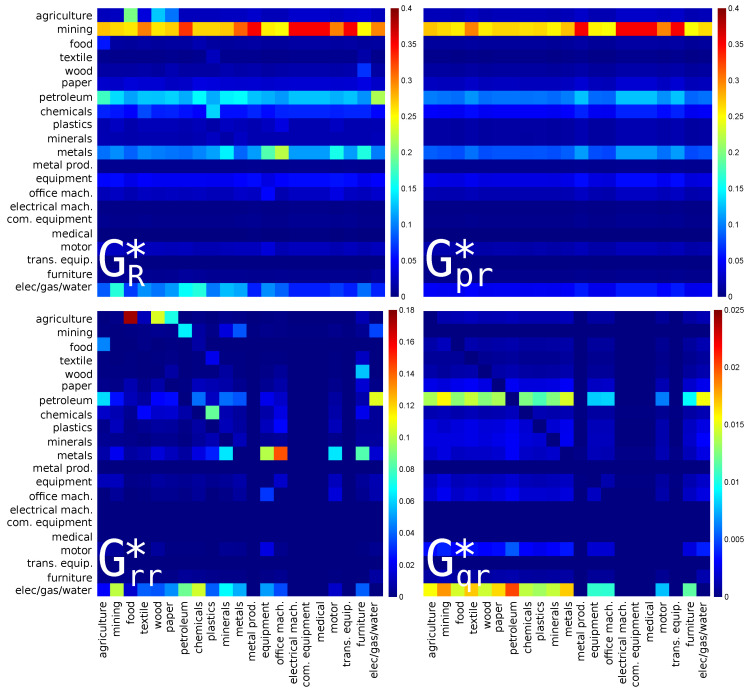
The same as in Figure 2 for Russia (RUS) in 2008. The corresponding matrix weights are: $W_{\mathrm{pr}}^{*}=0.804255$, $W_{\mathrm{rr}}^{*}=0.159634$, $W_{\mathrm{qr}}^{*}=0.036111$ and $W_{\mathrm{qrnd}}^{*}=0.033377$. For each panel, each cell corresponds to a given value of the Google matrix component ($G_{R}^{*}$, $G_{\mathrm{pr}}^{*}$, $G_{\mathrm{rr}}^{*}$, or $G_{\mathrm{qr}}^{*}$), the colorbar gives the correspondence between matrix elements values and colors (from blue for 0 to red for the maximum). This value characterizes the intensity of the interaction between two economical sectors. The direction of the interaction is from bottom to left.

The reduced Google matrices for Russia for PageRank (import) and CheiRank (export) directions are shown in Figure 3 and Figure 4 respectively. They are constructed in the same manner as Figure 1 and Figure 2 for USA.

For the reduced Google matrix $G_{R}$ of Russian economic sectors with PageRank (import) direction, see Figure 3, the strongest link is between **agriculture** and **food**. This is similar to previous depicted case of USA. We note here that the inverted link, i.e., from **food** to **agriculture**, is weaker but also present in $G_{R}$ and in $G_{\mathrm{rr}}$. This is certainly due to the fact that the food industry also produces products for animal used in agriculture. In $G_{\mathrm{pr}}$, the most pronounced horizontal line is for **food**, highlighting the fact that this industry uses indirectly products of almost all the other economic sectors; it is followed by the line of **motor** (s=18, manufacture of motor vehicles) and **elec/gas/water** (s=21). Among indirect links, the strongest one is from **minerals** (s=10, manufacture of other non-metallic mineral products) to **elec/gas/water**.

For the reduced Google matrix $G_{R}^{*}$ of the Russian economic sectors with CheiRank (export) direction, see Figure 4, there is the dominance of lines related to **mining**, followed, with weaker intensities, by **petroleum**, **metals**, and **elec/gas/water**. This picture is rather different from the USA case in Figure 2. Although there are only very few weak direct links pointing to **mining**, the mining sector is very important since, through the network of exports, it strongly acts (via almost only indirect interactions) upon every sector of the Russian economy. For the hidden links encoded in $G_{\mathrm{qr}}^{*}$, the dominant line is for **elec/gas/water**.

### 3.3. Interdependence of the Chinese Economy Sectors

**Figure 5:**
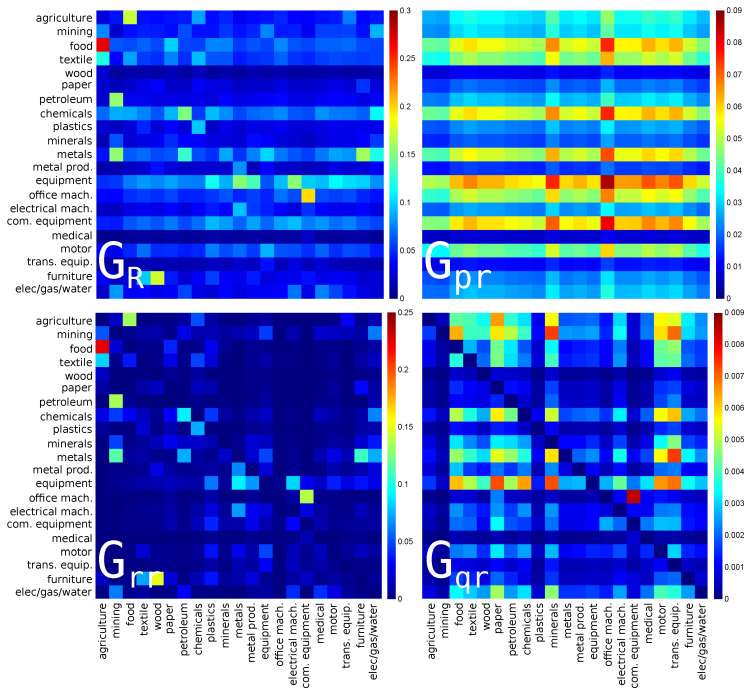
Same as in Figure 1 for China CHN in 2008. The corresponding matrix weights are: $W_{\mathrm{pr}}=0.698164$, $W_{\mathrm{rr}}=0.263683$, $W_{\mathrm{qr}}=0.038153$ and $W_{\mathrm{qrnd}}=0.035547$. For each panel, each cell corresponds to a given value of the Google matrix component ($G_{R}$, $G_{\mathrm{pr}}$, $G_{\mathrm{rr}}$, or $G_{\mathrm{qr}}$), the colorbar gives the correspondence between matrix elements values and colors (from blue for 0 to red for the maximum). This value characterizes the intensity of the interaction between two economical sectors. The direction of the interaction is from bottom to left.

**Figure 6:**
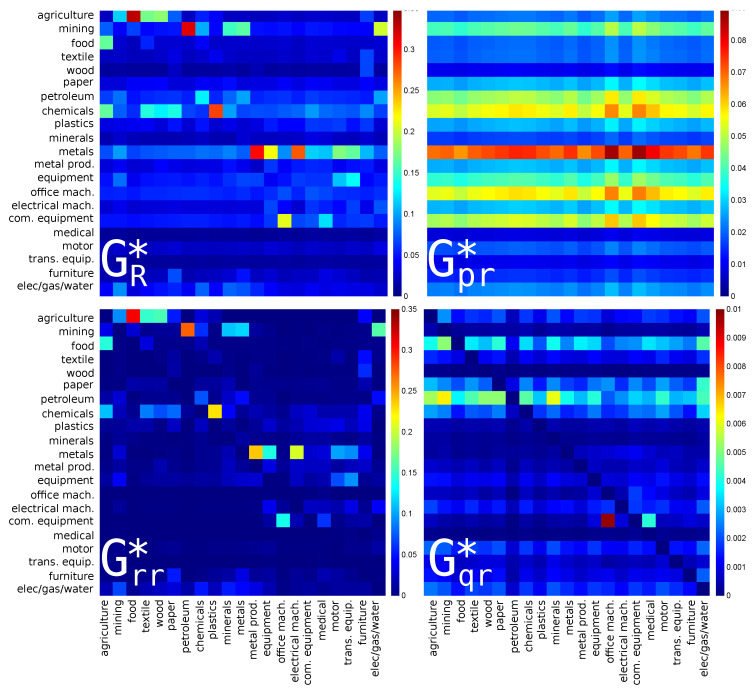
The same as in Figure 2 for China (CHN) in 2008. The corresponding matrix weights are: $W_{\mathrm{pr}}^{*}=0.647087$, $W_{\mathrm{rr}}^{*}=0.326402$, $W_{\mathrm{qr}}^{*}=0.026511$ and $W_{\mathrm{qrnd}}^{*}=0.024648$. For each panel, each cell corresponds to a given value of the Google matrix component ($G_{R}^{*}$, $G_{\mathrm{pr}}^{*}$, $G_{\mathrm{rr}}^{*}$, or $G_{\mathrm{qr}}^{*}$), the colorbar gives the correspondence between matrix elements values and colors (from blue for 0 to red for the maximum). This value characterizes the intensity of the interaction between two economical sectors. The direction of the interaction is from bottom to left.

Interdependencies of the economy sectors of China for PageRank (import) and CheiRank (export) directions are presented with the reduced Google matrix and its components in Figure 5 and Figure 6 respectively.

For the reduced Google matrix $G_{R}$, shown in Figure 5, there are strong links between **agriculture** and **food** similarly to the USA and Russian cases. In addition, there is a strong transition from **com. equipment** (s=16, manufacture of communication equipment) to **office mach.** (s=14, manufacture of computing machinery). This corresponds to strong Chinese production of TV, computers and other communication related products. In the matrix component $G_{\mathrm{pr}}$, there are strong lines for **food**, **chemicals**, **equipment** (machinery and equipment), and **com. equipment**. We note that, contrary to the USA and Russia, the food sector does not dominate alone as top importer. Indeed, chemistry, communication, computing, and machinery industries also play important roles as they also indirectly use products of many Chinese economic sectors. For the hidden links in the matrix component $G_{\mathrm{qr}}$, the strongest matrix element points from **com. equipment** to **office mach.**. Many other links, with a slightly weaker intensity, are also highlighted by the $G_{\mathrm{qr}}$ matrix component, but these are quite weak in comparison with the highest intensities in the reduced Google matrix $G_{R}$.

For the reduced Google matrix $G_{R}^{*}$, shown in Figure 6, the strongest matrix elements are from **food** to *agriculture*, from **metal prod.** to **metals**, from **petroleum** to **mining**, from **plastics** to **chemicals**, and from **electrical mach.** (electrical machinery and apparatus) to **metals**. For the $G_{\mathrm{pr}}^{*}$ matrix, the strongest horizontal line of transitions is for **metals**. Among the indirect matrix elements of $G_{\mathrm{qr}}^{*}$, the strongest link is from **office mach.** to **com. equipment**.

### 3.4. Intersensitivity of the Economy Sectors

The above results show specific dependencies between economy sectors for USA, Russia, and China. Here we choose 10 countries (USA, RUS, CHN, DEU, FRA, ITA, GBR, JAP, KOR, IND), for which we determine the balance sensitivity of each economical activity sectors to a price variation of a specific sector. Excepting KOR, these countries are among the top 10 importers according to PageRank algorithm applied to the global Google matrix *G*. KOR is ranked at the 12th position.

For a given country, the balance sensitivity of a sector $s^{\prime }$ to an infinitesimal increase of sector *s* product prices is $D\left(cs\to cs^{\prime }\right)=dB_{cs^{\prime }}/d\delta$. The balance of the economic sector *s* is defined as $B_{cs}=\left(P_{cs}^{*}-P_{cs}\right)/\left(P_{cs}^{*}+P_{cs}\right)$, see Section 2.4 for details.

**Figure 7:**
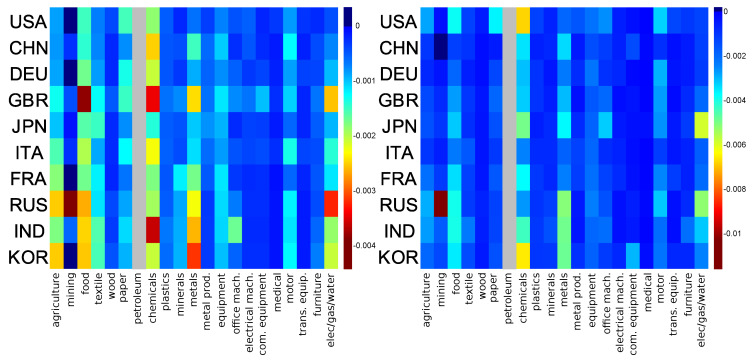
Sector balance sensitivity to **petroleum** sector for year 1995 (**left**) and 2008 (**right**); horizontal axis represents the sector index in data order; vertical axis represents the country index in PageRank order for the given year. For each couple (s,c) we modify the link from (**petroleum**, *c*) towards (s,c) and compute the (s,c) balance sensitivity, D(cpetroleum,cs). For each plot, a given color corresponds to a given intensity of the sensitivity D(cpetroleum,cs). Grey column represents self sensitivity (not shown).

**Figure A1:**
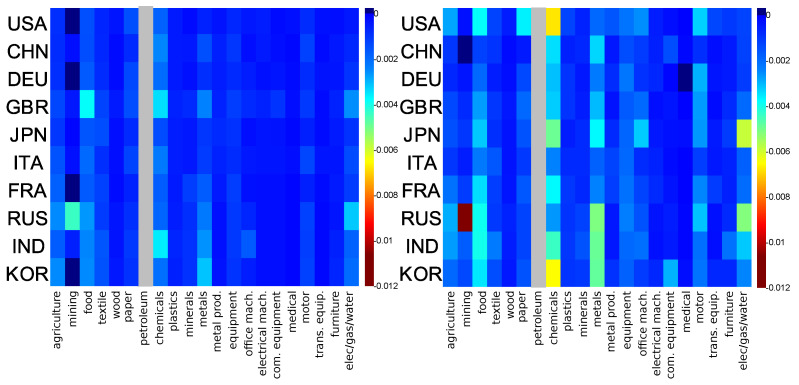
Same as Figure 7 but with same color scale for both panels. Sector balance sensitivity to **petroleum** sector for year 1995 (left) and 2008 (right); horizontal axis represents the sector index in data order; vertical axis represents the country index in PageRank order for the given year. For each couple (s,c) we modify the link from (**petroleum**, *c*) towards (s,c) and compute the (s,c) balance sensitivity, D(cpetroleum,cs). For each plot, a given color corresponds to a given intensity of the sensitivity D(cpetroleum,cs). Grey column represents self sensitivity (not shown).

In Figure 7, we show a map of the balance sensitivity to the **petroleum** sector for years 1995 and 2008. The maximal absolute value of the balance sensitivity *D* is increased approximately by a factor 3 from 1995 to 2008 showing an increased dependence of economy sectors to petroleum. Partially, this can be attributed to the petroleum price growth from 1995 to 2008 (changed by a factor ∼3, even ∼5 taking April 2008 as reference). For both years and for any of the considered countries, we observe that some economic sectors, such as **electrical mach.** (electrical machinery and apparatus), **medical** (medical, precision and optical instruments, watches and clocks), **trans. equip.** (transport equipment), and **wood**, are almost insensitive to the petroleum products sector. Inversely, the most sensitive economic sectors to **petroleum** sector are **chemicals**, **metals**, **elec/gas/water**, **food**, and (mostly in 1995) **agriculture**. Indeed, the activities of these industries directly use petroleum products. For each country, the **mining** sector is robust from 1995 to 2008 except for the Russian mining sector for which the balance sensitivity goes from −0.0045 in 1995 to −0.012 in 2008. This is a peculiarity of the Russian mining sector which appears strongly dependent on the Russian petroleum sector. The same data as in Figure 7 are represented in Figure A1 (see Appendix A) but with the same color scale for 1995 and 2008. In Figure A1, we globally observe that from 1995 to 2008 the **chemicals** sector increased its sensitivity to **petroleum** sector, by, e.g., a factor ∼3 for USA and KOR. A weaker increase of sensitivity to **petroleum** sector could be observed for the **metals** and **food** sectors. We also observe that Russian and Japanese **elec/gas/water** sectors become more sensitive to their national **petroleum** sector from 1995 to 2008. From Figure A1, we additionally note that all the economic sectors of Germany, Italy, and, to a somewhat less extent, of France, Great-Britain, and China remain insensitive to their **petroleum** economic sector from 1995 to 2008.

**Figure 8:**
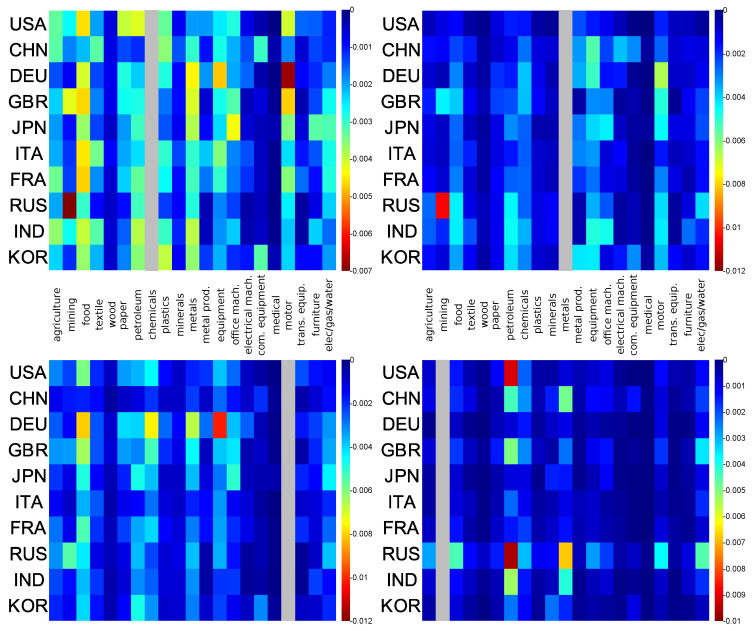
Sector balance sensitivity to **chemicals** (**top left**), **metals** (**top right**), **motor** (**bottom left**), and **mining** (**bottom right**) sectors for year 2008; horizontal axis represents the sector index in data order; vertical axis represents the country index in PageRank order for the given year. For each couple (s,c) we modify the link from $\left(s^{\prime },c\right)$ towards (s,c) and compute the (s,c) balance sensitivity, $D(cs^{\prime },cs)$. For each panel, a given color corresponds to a given intensity of the represented sensitivity. Grey column represents self sensitivity (not shown).

The 2008 sector balance sensitivities to **chemicals**, **metals**, **motor**, and **mining** sectors are shown in Figure 8. Among these economic sectors, the **chemicals** sector has the broadest impact on the other economic sectors. The strongest sensitivities to the **chemicals** and the **metals** sectors concern the Russian **mining** and the German **motor** sectors. The German economy is the most affected by the **motor** sector, particularly the **equipment**, the **food**, and the **chemicals** sectors. The most sensitive economic sectors to the **mining** sector are the **petroleum** and the **metals** sectors, particularly the Russian **petroleum** and **metals** sectors and the US **petroleum** sector.

### 3.5. Reduced Network of Economic Sectors

We construct the reduced networks of the 21 economic sectors for different countries. For that purpose, we use the import reduced Google matrices $G_{R}$ and export reduced Google matrix $G_{R}^{*}$ corresponding to the USA, Russia, and China economic sectors for 2019. Examples of such reduced Google matrices are presented in Section 3.1, Section 3.2 and Section 3.3 for 2008.

For a given country *c* and for the economic sector *s*, we select the four links ${\left\{\left(c,s\right)\to \left(c,s_{i}\right)\right\}}_{i=\alpha ,\beta ,\gamma ,\delta }$ giving the strongest entries in the composite matrix $G_{\mathrm{rr}}+G_{\mathrm{qrnd}}$ (or $G_{\mathrm{rr}}^{*}+G_{\mathrm{qrnd}}^{*}$) extracted from the reduced Google matrix $G_{R}$ (or $G_{R}^{*}$) associated to the 21 economic sectors ${\left\{\left(c,s_{i}\right)\right\}}_{i=1,\cdots ,21}$ (see Figure 1, Figure 2, Figure 3, Figure 4, Figure 5 and Figure 6 to have an idea of the composite matrices, $G_{\mathrm{rr}}+G_{\mathrm{qrnd}}$ and $G_{\mathrm{rr}}^{*}+G_{\mathrm{qrnd}}^{*}$, for USA, Russia, and China). The reduced networks constructed from the components of the reduced Google matrix $G_{R}$ ($G_{R}^{*}$) highlight import (export) capabilities of the economic sectors. Let us note that the compact picture given by the reduced Google matrices at the level of a country comprises in fact the global information encoded in the global Google matrix of all the transactions from any sector *s* of a country *c* to any sector $s^{\prime }$ of a country $c^{\prime }$.

**Figure 9:**
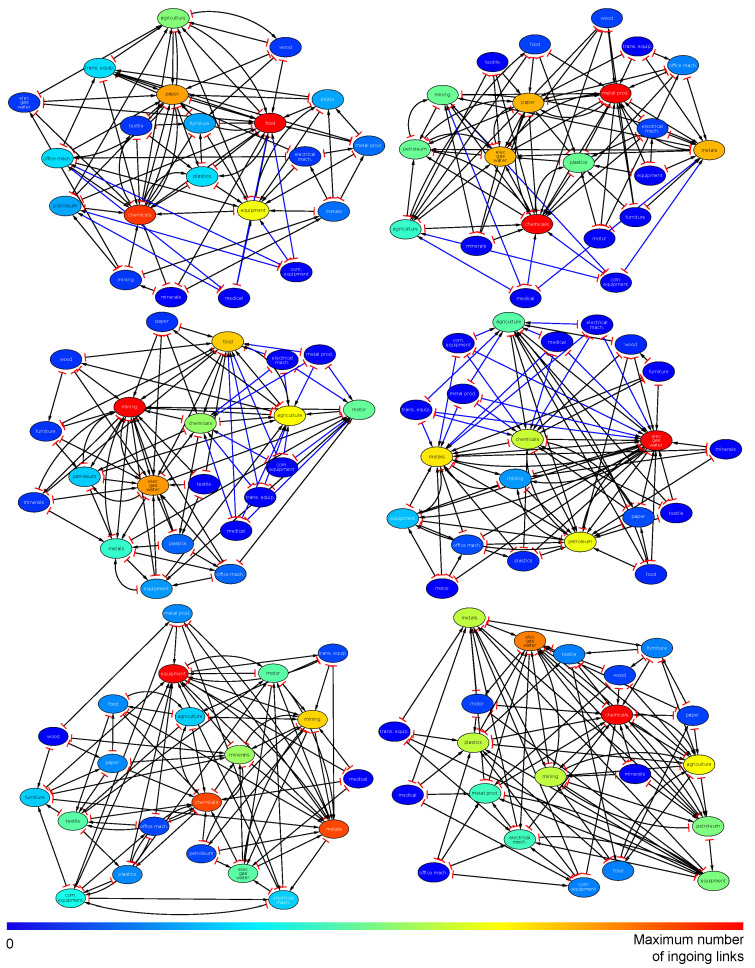
Reduced networks of economic sectors of the USA (**top row**), RUS (**central row**), and CHN (**bottom row**) obtained from the corresponding import reduced Google matrices $G_{R}$ (**left panel**) and export reduced Google matrices $G_{R}^{*}$ (**right panel**) for year 2009. For each country, the reduced networks were computed for a set of 21 major economic sectors. From each of them we drew the four strongest outgoing links. Node labels are sector codes from Table 1. The color of a node corresponds to its number of ingoing links from 0 (blue color) to the maximum (red color). We distinguish by the blue color hidden links from direct links present in the raw data. Red bars represent source-side of the links and arrows represent target-side of the links. The networks have been plotted with radial plot algorithm in Cytoscape software [42] with manual layout optimization.

In Figure 9 (top row) we present the reduced network of US economic sectors for import (left panel) and export (right panel) exchanges. From the import point of view, we observe that the **chemicals** sector uses products from the broadest variety of US economic sectors as it has the maximum of ingoing links (13 out of 21 economic sector are pointing to the **chemicals** sector). Other economic sectors using many US resources are **food** (10 out of 21), **paper** (10 out of 21), **equipment** (9 out of 21), and **agriculture** (7 out of 21) sectors. From the export point of view, we observe that the major suppliers of the US economic sectors are (by number of ingoing links) **chemicals** (13 out of 21 economic sectors are supplied by **chemicals** sector), **metal prod.** (13 out of 21), **elec/gas/water** (10 out of 21), and **metals** (9 out of 21). The **chemicals** sector seems to play an important role since it is an economic hub using products of many other economic sectors and being a supplier of many other economic sectors. From both the import and export pictures, we observe that the manufacture of equipment sectors, **com. equipment** (radio, television and communication equipment) and **medical** (medical, precision and optical instruments, watches and clocks) are linked to other economic sectors only by hidden links, i.e., in WNEA there is no direct commodities exchange between these sectors and the others.

In Figure 9 (middle row), we present the reduced network of the Russian economic sectors for import (left panel) and export (right panel) exchanges. Here the major importers are the following economic sectors: **mining** (18 out of 21), **elec/gas/water** (12 out of 21), **food** (11 out of 21). We note that the **mining** sector uses products of almost all the 21 considered sectors. From the export point of view, the major exporters are the sectors of **elec/gas/water** (21 of 21), **metals** (13 out of 21), **petroleum** (13 out of 21), **chemicals** (12 of 21), and **agriculture** (9 out of 21). We note that the **elec/gas/water** sector, which exploits products of all the other economic sectors, is very central in the Russian economy since it constitutes the major economic hub. From both import and export pictures, as in the US economy, the **medical** and **com. equipment** sectors are linked to the other by hidden links. In addtion to these sectors, the **electrical mach.** (electrical machinery), **metal prod.** (fabricated metal products), **trans. equip.** (transport equipment) sectors also intervene through hidden links.

In Figure 9 (bottom row), we present the reduced network of Chinese economic sectors for import (left panel) and export (right panel) exchanges. The major importers are the sectors of **equipment** (11 out of 21), **chemicals** (10 out of 21), **metals** (10 out of 21),and **mining** (8 out of 21). The major exporters are the sector of **chemicals** (12 out of 21), and **elec/gas/water** (10 out of 21). As in the US economy, the **chemicals** sector is an economic hub playing a central role in the Chinese economy.

### 3.6. Sensitivity of the EU Countries to the Petroleum Products Price

**Figure 10:**
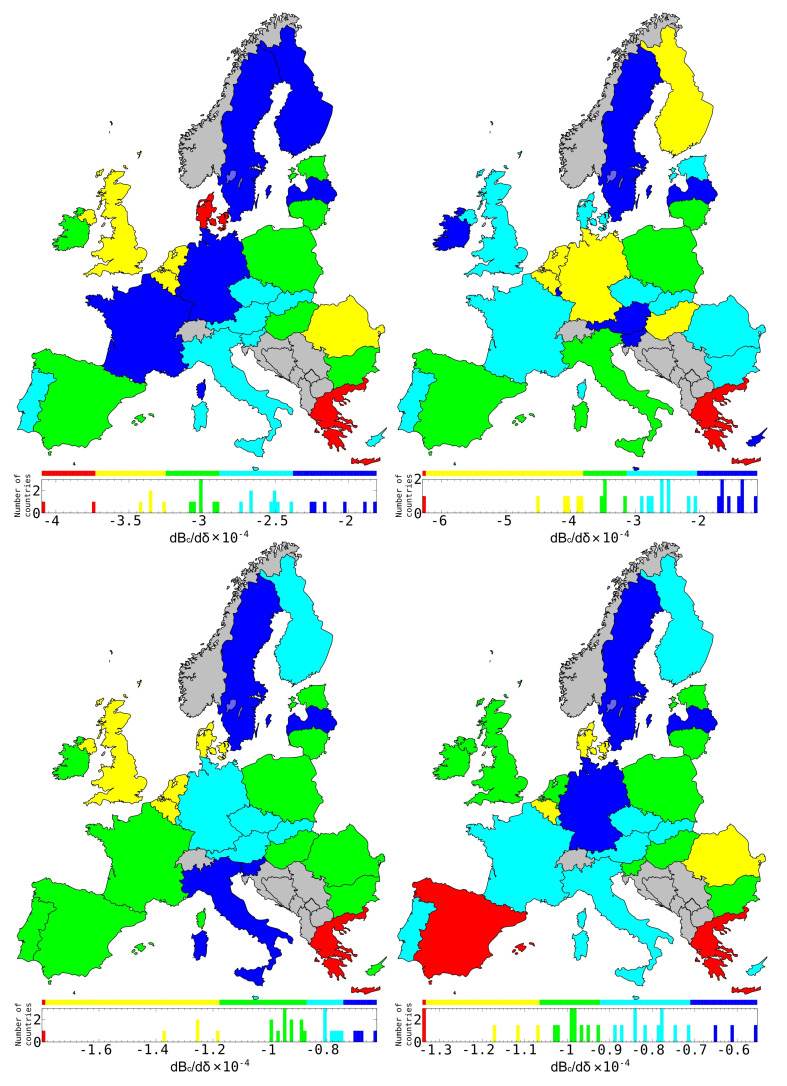
Balance sensitivity of the 27 EU countries in 2008 to export variation of the **petroleum** sector of USA (**top left**), RUS (**top right**), Norway (NOR) (**bottom left**) and Saudi Arabia (SAU) (**bottom right**). Color categories are obtained using the Jenks natural breaks classification method [43].

The combination of the WNEA data and the REGOMAX approach allow us to study the sensitivity of the country balance to a specific economy sector. In recent studies of the WTN from the EU COMTRADE database [22], such a sensitivity has been determined for the 27 EU countries (EU members in 2013) in respect to petroleum price variation. Here, for comparison, we show the balance sensitivity of the same 27 EU countries in respect to the price variation of the petroleum. The results are presented in Figure 10 for the **petroleum** sectors of USA, Russia, Norway and Saudi Arabia in 2008. We see that the most sensitive country to US, Russian, and Norwegian petroleum is Greece while the most sensitive to Saudian petroleum are Greece and Spain. Globally, the influence of the USA and Russia are comparable while the influences of Norway and Saudi Arabia (SAU) are by a factor 2–3 smaller.

We note that color maps of the EU balance sensitivities to petroleum products from USA, RUS, and SAU are somewhat different from the one obtained for the WTN case shown in [22] (Figure 6 middle row panels). We attribute this difference to the fact that the **petroleum** sector contains different petroleum related ISIC products while for the WTN only the petroleum product was considered. In addition, WNEA comprises real inter-sector and inter-country economic exchanges. We nevertheless note that the petroleum sensitivity of Netherlands is in any case moderate to strong as in the WTN study [22].

In Figure 10, we observe that Sweden, Finland, and Latvia are the less sensitive to petroleum products of any of the considered suppliers. In addition to these countries, we see that the less sensitive to petroleum products from US are France and Germany, from Russia are Austria, Slovenia and Ireland, from Norway is Italy, and from Saudi Arabia is Germany. In addition to Greece which is the most sensitive country to petroleum products for any of the considered suppliers, the most sensitive are Denmark to US petroleum products, and Spain to Saudian petroleum products.

## 4. Discussion

In this work, we apply the reduced Google matrix (REGOMAX) analysis to the World Network of Economic Activities (WNEA) data in order to determine the interdependence of the economy activity sectors for several countries with the main accent on USA, Russia, and China. There are similarities and significant differences for the interactions of the economy sectors of the selected countries. All the three countries exhibit strong interdependence between **agriculture** and **food** sectors, that is rather natural since all people need agriculture development for food productions, and also between **mining** and **petroleum** sectors, that is also very natural. For the US economy, we note that there are also strong interdependence between **metals** and **metal prod.** sectors, and between **plastics**, **textile**, and **chemicals** sectors. For the Chinese economy, we observe additional interdependence between **com. equipment** and **office mach.**, and **electrical mach.** to **metals**. From the constructed reduced networks of economic sectors, for each considered economy, we have determined an economic hub which uses a broad variety of products from the other economic sectors and supplies also many of them. For the US economy, the **chemicals** sector is clearly an economic hub. For the Russian economy, the **elec/gas/water** sector is central as it is the main exporters to all the other sectors and in return this sector also consumes many resources from the other sectors. For the Chinese economy, as for the US, the **chemicals** sector is an economic hub. We also determine the sensitivity of the sectors of a given country to the variation of the price of a specific sector. Globally, for any of the top importer countries according to PageRank algorithm applied to the WNEA (USA, RUS, CHN, DEU, FRA, ITA, GBR, JAP, KOR, IND), we observe a strong sensitivity of the **chemicals**, **metals**, **elec/gas/water**, and **food** sectors to the price increase of the **petroleum** sector products. Contrarily, the **electrical mach.**, **com. equipment**, **trans. equip.**, and **wood** sectors are the most insensitive to the **petroleum** sector. We compute also the sensitivities of the economic sectors to **chemicals**, **metals**, **motor**, and **mining** sectors and we determine the color map of the EU countries balance sensitivities to a price increase of products from US, Russian, Norwegian, and Saudian **petroleum** sectors.

Our study demonstrates that the REGOMAX method allows us to find interdependencies between economy sectors for selected countries. The WNEA data of OECD-WTO contains transformations of production of one sector to another that is absent for multiproduct trade data of COMTRADE. Thus, it would be very desirable to extend OECD-WTO data for more sectors and more recent years. We hope that this will happen in future years.

## Figures and Tables

**Table 1:** List of sectors considered by input/output matrices from the WTO-OECD database, their correspondence to the ISIC UN classification is also given. The second column gives the short names we use in the present paper to designate the different economical sectors.

*s*	Short Name	OECD ICIO Category	ISIC Rev. 3 Correspondence
1	**agriculture**	C01T05 AGR	01—Agriculture, hunting and related service activities
			02—Forestry, logging and related service activities
			05—Fishing, operation of fish hatcheries and fish farms; service activities incidental to fishing
2	**mining**	C10T14 MIN	10—Mining of coal and lignite; extraction of peat
			11—Extraction of crude petroleum and natural gas; service activities incidental to oil and gas extraction excluding surveying
			12—Mining of uranium and thorium ores
			13—Mining of metal ores
			14—Other mining and quarrying
3	**food**	C15T16 FOD	15—Manufacture of food products and beverages
			16—Manufacture of tobacco products
4	**textile**	C17T19 TEX	17—Manufacture of textiles
			18—Manufacture of wearing apparel; dressing and dyeing of fur
			19—Tanning and dressing of leather; manufacture of luggage, handbags, saddlery, harness and footwear
5	**wood**	C20 WOD	20—Manufacture of wood and of products of wood and cork, except furniture;
			Manufacture of articles of straw and plaiting materials
6	**paper**	C21T22 PAP	21—Manufacture of paper and paper products
			22—Publishing, printing and reproduction of recorded media
7	**petroleum**	C23 PET	23—Manufacture of coke, refined petroleum products and nuclear fuel
8	**chemicals**	C24 CHM	24—Manufacture of chemicals and chemical products
9	**plastics**	C25 RBP	25—Manufacture of rubber and plastics products
10	**minerals**	C26 NMM	26—Manufacture of other non-metallic mineral products
11	**metals**	C27 MET	27—Manufacture of basic metals
12	**metal prod.**	C28 FBM	28—Manufacture of fabricated metal products, except machinery and equipment
13	**equipment**	C29 MEQ	29—Manufacture of machinery and equipment n.e.c.
14	**office mach.**	C30 ITQ	30—Manufacture of office, accounting and computing machinery
15	**electrical mach.**	C31 ELQ	31—Manufacture of electrical machinery and apparatus n.e.c.
16	**com. equipment**	C32 CMQ	32—Manufacture of radio, television and communication equipment and apparatus
17	**medical**	C33 SCQ	33—Manufacture of medical, precision and optical instruments, watches and clocks
18	**motor**	C34 MTR	34—Manufacture of motor vehicles, trailers and semi-trailers
19	**trans. equip.**	C35 TRQ	35—Manufacture of other transport equipment
20	**furniture**	C36T37 OTM	36—Manufacture of furniture; manufacturing n.e.c.
			37—Recycling
21	**elec/gas/water**	C40T41 EGW	40—Electricity, gas, steam and hot water supply
			41—Collection, purification and distribution of water
22	**construction**	C45 CON	45—Construction
23	**vehicles**	C50T52 WRT	50—Sale, maintenance and repair of motor vehicles and motorcycles; retail sale of automotive fuel
			51—Wholesale trade and commission trade, except of motor vehicles and motorcycles
			52—Retail trade, except of motor vehicles and motorcycles; repair of personal and household goods
24	**hotels/restaurants**	C55 HTR	55—Hotels and restaurants
25	**transport**	C60T63 TRN	60—Land transport; transport via pipelines
			61—Water transport
			62—Air transport
			63—Supporting and auxiliary transport activities; activities of travel agencies
26	**telecom**	C64 PTL	64—Post and telecommunications
27	**financial**	C65T67 FIN	65—Financial intermediation, except insurance and pension funding
			66—Insurance and pension funding, except compulsory social security
			67—Activities auxiliary to financial intermediation
28	**real estate**	C70 REA	70—Real estate activities
29	**renting equip.**	C71 RMQ	71—Renting of machinery and equipment without operator and of personal and household goods
30	**computer**	C72 ITS	72—Computer and related activities
31	**R&D**	C73 RDS	73—Research and development
32	**other1**	C74 BZS	74—Other business activities
33	**public**	C75 GOV	75—Public administration and defense; compulsory social security
34	**education**	C80 EDU	80—Education
35	**health**	C85 HTH	85—Health and social work
36	**other2**	C90T93 OTS	90—Sewage and refuse disposal, sanitation and similar activities
			91—Activities of membership organizations n.e.c.
			92—Recreational, cultural and sporting activities
			93—Other service activities
37	**private**	C95 PVH	95—Private households with employed persons
